# D-dimer levels in acute exacerbation of asthma among children: A correlation study

**DOI:** 10.6026/973206300214791

**Published:** 2025-12-15

**Authors:** Pragya Pragya, Prateek Pandey, Ankita Dinkar, Fahad Naaz, Ashutosh Kumar, Partha Kumar Chaudhuri

**Affiliations:** 1 Department of Paediatrics, Rajendra Institute of Medical Sciences, Ranchi, Jharkhand, India; 2 Department of Paediatrics, Sheikh Bhikhari Medical College and Hospital, Hazaribagh, Jharkhand, India

**Keywords:** D-dimer, asthma exacerbation, children, coagulation abnormalities, biomarker, pulmonary score

## Abstract

Asthma exacerbations in children may involve systemic coagulation changes reflected by elevated D-dimer levels. Therefore, it is of interest to evaluate 50 children aged 2-15 years to assess the correlation between D-dimer levels and acute exacerbation severity. D-dimer positivity increased significantly with severity-from 9.5% (mild) to 45.5% (moderate) and 71.4% (severe) (p=0.004). Positive D-dimer results were also strongly associated with longer hospital stays (p<0.001). Thus, we show that D-dimer is a valuable biomarker for assessing exacerbation severity and predicting clinical outcomes in pediatric asthma.

## Background:

Asthma is a chronic inflammatory respiratory disease characterized by airway hyper responsiveness, reversible airflow obstruction and variable respiratory symptoms [1]. It affects approximately 14% of children globally, with significant regional variations and increasing prevalence in low- and middle-income countries [2]. Acute exacerbations of asthma represent a major clinical challenge, particularly in pediatric populations, where they can lead to emergency department visits, hospitalizations and in severe cases, life-threatening respiratory failure [3]. Recent research has revealed that asthma exacerbations involve complex systemic inflammatory responses that extend beyond simple airway obstruction [4]. These episodes may activate coagulation pathways, leading to a prothrombotic state characterized by elevated levels of fibrin degradation products such as D-dimer [5]. D-dimer, a specific degradation product of cross-linked fibrin, is widely used as a biomarker for thrombotic disorders but has recently gained attention for its potential role in inflammatory conditions including asthma [6]. The relationship between asthma and coagulation abnormalities has been increasingly documented in the literature. Studies have shown that asthma exacerbations can trigger the release of tissue factor, initiating the extrinsic coagulation cascade and resulting in thrombin generation and fibrin deposition in the airways [7]. This hyper coagulable state may contribute to airway inflammation, remodeling and increased risk of thromboembolic complications [8]. In adult populations, elevated D-dimer levels during asthma exacerbations have been associated with increased disease severity, prolonged hospitalization and higher risk of adverse outcomes [9, 10- 11]. Despite these findings, substantial gaps remain in our understanding of the relationship between D-dimer levels and asthma exacerbation severity in children. Most studies have focused on adult populations or have included small sample sizes, limiting the generalizability of their findings [12]. Additionally, the potential utility of D-dimer as a prognostic biomarker for clinical outcomes such as duration of hospitalization and oxygen requirements in pediatric asthma exacerbations has not been thoroughly investigated [13]. The complex interplay between inflammation and coagulation in asthma pathophysiology warrants further investigation, particularly in pediatric populations where the disease burden is substantial [14]. Understanding this relationship could lead to improved risk stratification, more targeted therapeutic interventions and better clinical outcomes for children with asthma exacerbations [15]. Therefore, it is of interest to establish a positive correlation between D-dimer levels and the severity of acute exacerbations of bronchial asthma in children aged 2-15 years.

## Materials and Methods:

## Study design and setting:

A prospective observational study was conducted over an 18-month period in the Department of Pediatrics at Rajendra Institute of Medical Sciences (RIMS), Ranchi, Jharkhand. The study was approved by the Institutional Ethics Committee and written informed consent was obtained from guardians of all participants before enrollment.

## Study population:

The study included children aged 2-15 years admitted to the Pediatrics Department with a diagnosis of acute exacerbation of bronchial asthma. The diagnosis of asthma and its exacerbation was based on clinical history, physical examination and response to bronchodilator therapy, following the Global Initiative for Asthma (GINA) guidelines.

## Sample size calculation:

The sample size was calculated based on the prevalence of childhood asthma in the pediatric population (15.7%). Using the formula for sample size calculation: n = (Z^2^.P.Q)/d^2^, where Z = 1.96 (for 95% confidence level), P = 15.7% (prevalence), Q = 84.3% (1-P) and d = 10% (relative allowable error), the calculated sample size was 50 participants.

## Inclusion and exclusion criteria:

Children were included if they met the following criteria: (1) age between 2-15 years; (2) diagnosed with acute exacerbation of bronchial asthma; and (3) admitted to the Pediatrics ward of RIMS, Ranchi.

Exclusion criteria included: (1) sepsis; (2) bleeding diathesis (qualitative or quantitative disorders); (3) children born to mothers with a history of placental insufficiency, eclampsia, preeclampsia, hypertension, HELLP syndrome, or use of platelet-lowering medications during pregnancy; (4) use of medications that alter the bleeding cascade; (5) lymphoreticular malignancies; and (6) autoimmune or alloimmune disorders.

## Data collection

A structured data collection form was used to record demographic information (age, gender), clinical details (episode of asthma exacerbation, severity, history of exacerbations) and laboratory test results (D-dimer levels). The severity of the current exacerbation was assessed using the pulmonary score from the Spanish Guide for Asthma Management (GEMA 5.0), which evaluates respiratory rate, wheezing and accessory muscle use, with scores ranging from 0 to 9.

## Laboratory procedures:

Blood samples (3 mL) were collected from each participant using a sterile syringe and serum analysis vacutainer. Samples were sent to the Pathology Department at RIMS for D-dimer estimation using the Standard F D-dimer FIA kit. The procedure involved collecting 10 µL of specimen using a standard Ezi tube, transferring it to extraction buffer, mixing 2-3 times, applying 100 µL of the specimen mixture to the specimen well of the test device and starting the test using an analyzer (F100, F200, or F2400). Results were displayed automatically after 7 minutes. D-dimer levels were interpreted as follows: below 25 ng/mL FEU (below measurable range), above 5,000 ng/mL FEU (above measurable range), with a reference range of <500 ng/mL FEU. Additional coagulation parameters including prothrombin time (PT), activated partial thromboplastin time (aPTT) and platelet count were measured using standard laboratory techniques. C-reactive protein (CRP) levels were also assessed as a marker of inflammation.

## Statistical analysis:

Data were entered into MS Excel and analyzed using the Statistical Package for the Social Sciences (SPSS) version 25.0. Both parametric and non-parametric tests were used based on the normality of the data. Descriptive statistics were presented as mean ± standard deviation (SD) for continuous variables and frequencies (percentages) for categorical variables. The chi-square test was used to assess associations between categorical variables, while the independent samples t-test or one-way ANOVA was used for continuous variables. A p-value <0.05 was considered statistically significant.

## Results:

The study included 50 children with acute exacerbation of bronchial asthma, with a mean age of 8.78 ± 3.4 years. The majority of participants were male (62%) and the largest age group was 6-10 years (46%). Most participants (80%) belonged to lower socioeconomic status. The mean weight and height of participants were 25 ± 9.56 kg and 117 ± 20 cm, respectively. A family history of asthma or allergies was reported by 76% of participants. The mean number of exacerbations in the past year was 2.72 ± 1.4. Regarding the severity of the current exacerbation, 42% were mild, 44% were moderate and 14% were severe. Exposure to passive smoking at home was reported by 40% of participants. The most common asthma management medication was inhaled corticosteroids (62%), followed by short-acting beta agonists (22%). The mean D-dimer level was 807 ± 1118 ng/mL, with a wide range (35-5000 ng/mL). The mean prothrombin time (PT) was 12.2 ± 1.99 seconds and the mean activated partial thromboplastin time (aPTT) was 30.3 ± 4.8 seconds. The mean platelet count was 329,973 ± 130,265 x10^9^/L. The mean C-reactive protein (CRP) level was 5.99 ± 4.7 mg/L. The mean duration of hospital stay was 3.58 ± 1.6 days. Oxygen therapy was required by 82% of participants. Significant association was found between D-dimer positivity and the severity of the current exacerbation (p=0.004). Among participants with mild exacerbations, only 9.5% had positive D-dimer levels, compared to 45.5% in the moderate group and 71.4% in the severe group (Table 1 - see PDF). A highly significant association was observed between D-dimer positivity and the duration of hospital stay (p<0.001). None of the participants with a 1-day or 2-day hospital stay had positive D-dimer levels, whereas the proportion of positive D-dimer results increased with longer hospital stays, reaching 100% in those with 7-day stays (Table 2 - see PDF). A statistically significant association was found between aPTT and family history of asthma or allergies (p=0.023). The mean aPTT was 27.6 ± 6.1 seconds in participants without a family history and 31.2 ± 4.0 seconds in those with a family history (Table 3 - see PDF).

## Discussion:

This study demonstrates a significant association between elevated D-dimer levels and the severity of acute asthma exacerbations in children, as well as with the duration of hospital stay. These findings suggest that D-dimer may serve as a valuable biomarker for assessing exacerbation severity and predicting clinical outcomes in pediatric asthma patients. The observed correlation between D-dimer levels and exacerbation severity (p=0.004) is consistent with previous research in both adult and pediatric populations. Our findings extend these observations to a pediatric population, highlighting the potential utility of D-dimer as a severity marker across age groups. The highly significant association between D-dimer positivity and prolonged hospital stay (p<0.001) represents a novel finding with important clinical implications [15, 16- 17]. To our knowledge, few studies have specifically examined the relationship between D-dimer levels and duration of hospitalization in pediatric asthma exacerbations. This association may reflect the underlying pathophysiological processes, where more severe inflammation leads to both greater coagulation activation and more prolonged recovery periods [18]. The finding that all patients with 7-day hospital stays had positive D-dimer levels suggests that this marker may help identify children at risk for prolonged hospitalization at an early stage, potentially allowing for more intensive monitoring and intervention. The significant association between aPTT and family history of asthma or allergies (p=0.023) is an intriguing finding that warrants further investigation. While the mean aPTT values remained within normal limits in both groups, the prolongation observed in children with a family history suggests a possible genetic predisposition to altered coagulation responses in these patients. The wide range of D-dimer levels observed in our study (35-5000 ng/mL) reflects the heterogeneity of asthma exacerbations and suggests that individual factors such as genetic predisposition, environmental triggers and comorbidities may influence the degree of coagulation activation. The lack of significant association between D-dimer levels and age, gender, or number of previous exacerbations in our study suggests that coagulation activation during asthma exacerbations may be more related to the intensity of the current inflammatory response rather than to demographic factors or asthma history. This finding contrasts with some previous research that has suggested age-related differences in coagulation parameters in respiratory diseases [19]. The discrepancy may be due to differences in study populations or methodologies, highlighting the need for further research in this area. The clinical implications of our findings are substantial. The correlation between D-dimer levels and exacerbation severity suggests that this biomarker could be incorporated into clinical assessment tools to improve risk stratification and guide treatment decisions. For example, children with elevated D-dimer levels might benefit from more aggressive anti-inflammatory therapy or closer monitoring for complications. Additionally, the association with prolonged hospital stay suggests that D-dimer testing at admission could help predict resource utilization and discharge planning. The potential role of antithrombotic agents in managing severe asthma exacerbations with elevated D-dimer levels represents an exciting avenue for future research. While our study was not designed to evaluate therapeutic interventions, the observed coagulation abnormalities suggest that antithrombotic therapy might be beneficial in selected patients. Previous studies have suggested that heparins, in addition to their anticoagulant effects, may have anti-inflammatory properties that could be beneficial in asthma [20]. However, the risk-benefit ratio of such interventions in pediatric populations requires careful evaluation through randomized controlled trials. Several limitations of our study should be acknowledged. First, the relatively small sample size (n=50) may have limited our ability to detect smaller associations or perform subgroup analyses. Second, the single-center design may limit the generalizability of our findings to other populations. Third, we did not assess long-term outcomes or the potential relationship between D-dimer levels during exacerbations and future asthma control or lung function. Finally, while we observed significant associations, our cross-sectional design does not allow us to establish causality or determine the temporal relationship between coagulation activation and exacerbation severity. Future research should address these limitations through larger, multicenter studies with longitudinal follow-up. Additionally, studies investigating the mechanisms linking inflammation and coagulation in asthma exacerbations could provide valuable insights for targeted therapeutic interventions. Finally, randomized controlled trials evaluating the safety and efficacy of antithrombotic agents in pediatric asthma exacerbations with elevated D-dimer levels are warranted.

## Conclusion:

A significant correlation between elevated D-dimer levels and the severity of acute asthma exacerbations in children aged 2-15 years. Additionally, D-dimer positivity was strongly associated with prolonged hospital stay, suggesting its potential utility as a prognostic biomarker. The significant association between aPTT and family history of asthma or allergies indicates a possible genetic predisposition to altered coagulation responses in these patients.
